# Sprouted Black Quinoa Extract Alleviates Heat Stress-Induced Liver Injury in Rats by Activating Nrf2 Signaling and Suppressing the NF-κB/NLRP3 Inflammasome Pathway

**DOI:** 10.3390/foods14162758

**Published:** 2025-08-08

**Authors:** Jing Zhou, Wenting Lv, Zhonghao Li, Li Wang, Bing Guo, Donghua Du

**Affiliations:** Department of Veterinary Medicine, College of Animal Science and Technology, Hebei North University, Zhangjiakou 075131, Chinawintonluce@163.com (W.L.); guobing810304@163.com (B.G.)

**Keywords:** heat stress, liver injury, black quinoa, oxidative stress, apoptosis, Nrf2 pathway, NF-κB/NLRP3 signaling

## Abstract

Heat stress (HS) is known to cause liver injury through mechanisms involving oxidative stress and inflammation, thereby highlighting the need for effective therapeutic interventions. This study evaluated the efficacy of sprouted black quinoa extract (SBQE) in mitigating HS-induced liver injury in a rat model. SBQE was obtained through an ultrasonication-assisted ethanol–water extraction process from black quinoa germinated for 48 h. Sprague Dawley rats (male) were administered via oral gavage SBQE at doses of 200, 400, or 800 mg/kg prior to each HS exposure (40 °C for 2 h per day over a period of 8 days). Pretreatment with SBQE resulted in a dose-dependent reduction in serum alanine aminotransferase (ALT) and aspartate aminotransferase (AST) levels, with the high dose (800 mg/kg) reducing these enzyme levels (*p* < 0.001 vs. HS group) and alleviating histopathological damage, including a significant decrease in hepatocyte vacuolization and inflammatory cell infiltration (histopathological scores were reduced by *p* < 0.001 in the 800 mg/kg SBQE group vs. HS group). SBQE also dose-dependently inhibited the accumulation of mitochondrial reactive oxygen species (mean fluorescence intensity decreased by *p* < 0.001 at 800 mg/kg) and the formation of malondialdehyde while restoring the activities of antioxidant enzymes such as superoxide dismutase (*p* < 0.01 at 800 mg/kg), catalase (*p* < 0.05 at 800 mg/kg), and glutathione peroxidase (*p* < 0.001 at 800 mg/kg), as well as replenishing glutathione levels (*p* < 0.001 at 800 mg/kg). Furthermore, the levels of proinflammatory cytokines (tumor necrosis factor-alpha, interleukin-6, interleukin-1β, and interleukin-18) in liver tissue were significantly reduced (with the high dose leading to *p* < 0.001 vs. HS group), which was associated with enhanced nuclear translocation of nuclear factor erythroid 2-related factor 2 (Nrf2; *p* < 0.05 at 800 mg/kg) and decreased phosphorylation of nuclear factor-κB p65 (NF-κB; *p* < 0.001 at 800 mg/kg). Additionally, the protein expression of NOD-like receptor pyrin domain-containing 3 (NLRP3) inflammasome components and markers of apoptosis were diminished. The results demonstrated that SBQE alleviated HS-induced liver injury by concurrently activating the Nrf2 antioxidant pathway and suppressing NF-κB/NLRP3 inflammasome signaling, suggesting its potential as a nutraceutical intervention for HS-related hepatotoxicity.

## 1. Introduction

Heat stress (HS), resulting from environmental or occupational exposure to elevated temperatures, represents a substantial risk to both human and animal health, leading to multi-organ dysfunction [1,2]. In humans, those working in agriculture, construction, and firefighting face increased risks of heat-related illnesses, such as heat exhaustion, heatstroke, and organ damage [1]. In animal husbandry, HS reduces livestock productivity, raises mortality rates, and causes economic losses, while also triggering inflammation and organ damage [2]. This highlights the urgent need for effective interventions to combat heat stress.

The liver, as a central organ for metabolism and detoxification, is especially susceptible to HS due to its high mitochondrial density and its critical role in thermoregulation [3,4]. The pathogenesis of HS-induced liver injury is closely associated with oxidative stress and dysregulated inflammatory responses [5]. Hyperthermia impairs mitochondrial electron transport, leading to the overproduction of reactive oxygen species (ROS), which surpass the capacity of endogenous antioxidant defenses [6]. This oxidative damage results in lipid peroxidation and the degradation of proteins and DNA, thereby directly contributing to hepatocyte apoptosis [7]. Simultaneously, ROS activate the transcription factor-κB (NF-κB), a key regulator of inflammatory processes [8]. The translocation of NF-κB to the nucleus promotes the expression of proinflammatory cytokines, such as tumor necrosis factor-alpha (TNF-α), interleukin-6 (IL-6), and interleukin-1β (IL-1β), as well as components of the inflammasome [8].

The NLRP3 inflammasome plays a critical role as a primary sensor of liver injury in both human HS cases and corresponding animal models. Upon activation, NLRP3 recruits apoptosis-associated speck-like protein containing a CARD (ASC) and procaspase-1, facilitating the autoactivation of caspase-1 [9]. The activated caspase-1 subsequently cleaves pro-IL-1β and pro-IL-18 into their mature, highly inflammatory forms and induces pyroptosis, a lytic form of programmed cell death [9,10]. This process results in an increased release of IL-1β and IL-18, which exacerbates inflammation and tissue damage, thereby establishing a self-perpetuating cycle [10,11]. Furthermore, oxidative stress and inflammatory cytokines collectively activate apoptotic pathways, underscoring the importance of apoptosis assessment in evaluating therapeutic interventions.

The Keap1–nuclear factor erythroid 2-related factor 2 (Nrf2)–antioxidant response element (ARE) pathway serves as a fundamental endogenous defense mechanism against oxidative stress [11]. Under normal physiological conditions, Nrf2 is retained in the cytoplasm by Keap1, which facilitates its proteasomal degradation. However, oxidative stress disrupts the Keap1-Nrf2 interaction, enabling Nrf2 to translocate into the nucleus. Within the nucleus, Nrf2 binds to the ARE, thereby promoting the transcription of cytoprotective genes that encode enzymes such as heme oxygenase-1 (HO-1), superoxide dismutase (SOD), catalase (CAT), and glutathione peroxidase (GSH-Px) [4,11]. Consequently, the activation of Nrf2 emerges as a promising therapeutic target for alleviating oxidative liver damage induced by heat stress.

Considering the limitations and adverse effects associated with synthetic drugs, natural compounds possessing antioxidant and anti-inflammatory properties present promising alternatives for hepatoprotection [12]. Black quinoa (Chenopodium quinoa Willd.), a nutrient-rich pseudocereal, is abundant in bioactive compounds, including polyphenols, flavonoids, saponins, vitamins, and essential amino acids [13,14]. Sprouting is a well-established bioprocessing technique that markedly enhances the bioavailability and concentration of these bioactive constituents, particularly antioxidants such as phenolic acids and flavonoids, through the activation of endogenous enzymes [15,16]. As a result, sprouted black quinoa (SBQ) demonstrates increased antioxidant and anti-inflammatory potential compared to its unsprouted form. Preliminary studies indicate that quinoa extracts exhibit hepatoprotective effects in models of chemical toxicity [17,18]; however, the specific effects of SBQ extract (SBQE) on HS-induced liver injury, along with the underlying mechanisms, have yet to be investigated.

Importantly, as far as we are aware, no research has yet examined whether SBQE simultaneously influences the Nrf2 antioxidant pathway and the NF-κB/NLRP3 inflammasome axis to confer protection against HS-induced hepatic damage and apoptosis. Consequently, this study investigated the protective effects and underlying mechanisms of SBQE in mitigating liver injury within a rat model of HS.

## 2. Materials and Methods

### 2.1. Preparation of SBQE

Black quinoa seeds (Quinoa Industry Development Company, Zhangjiakou, China) underwent sterilization using a 1% sodium hypochlorite (NaClO) solution for a duration of 10 min. Subsequently, the seeds were rinsed and placed in Petri dishes lined with two sheets of Whatman filter paper, which were moistened with 2 mL of distilled water. The Petri dishes were then incubated in complete darkness at a temperature of 37 °C for 48 h. The sprouted seeds were dried in an oven at 45 °C and then ground into a fine powder and sieved through an 80-mesh sieve [19].

The extraction procedure for SBQE was adapted from the method described by Arlene et al. [20], with certain modifications. In summary, SBQ powder was combined with an ethanol–water solvent in an 80:20 volume-to-volume ratio and subjected to probe ultrasonication at 37 Hz and 100% intensity for 15 min at 40 °C. Following ultrasonication, the mixture was centrifuged at 6000× *g* for 30 min, and the supernatant was collected. The solvent was then removed using a rotary vacuum evaporator set at 40 °C with an evaporation rate of 4 g/min. The resulting residue was dried at 50 °C to yield a powder. The SBQE extract was subsequently stored at −18 °C until further use. The extraction yield of SBQE was calculated as [X] mg of dried extract per gram of sprouted black quinoa powder (mean ± standard deviation, *n* = 3), where [X] is the actual yield obtained from our extraction experiments.

### 2.2. HS Protocol in Rats

Sprague Dawley (SD) rats, with an average weight of 200 ± 20 g, aged 6 weeks, and all male, were procured from SIBEIFU Biotechnology Co., Ltd., located in Beijing, China. Upon arrival, the rats underwent a 7-day acclimatization period under standard laboratory conditions, which included a temperature of 21 ± 1 °C, a 12 h light/dark cycle, and unrestricted access to food and water. All experimental procedures were reviewed and approved by the Scientific Research and Ethics Committee of Hebei North University. The HS protocol was implemented as previously described in our study [21]. In summary, the rats were exposed to a climate-controlled chamber (BX-200; Shanghai Boxun Industrial Co., Ltd., Shanghai, China) set at 40 ± 0.2 °C with a relative humidity of 60–65% for 2 h daily, from 10:00 a.m. to 12:00 p.m., over a span of 8 consecutive days. After each HS session, the rats were immediately returned to their standard housing conditions.

### 2.3. Animal Experimental Design

A total of thirty-six rats were randomly assigned to six groups, with each group comprising six rats (*n* = 6). SBQE was dissolved in 2 mL of distilled water for administration. Rats in the control group received equivalent volumes of distilled water (without HS) via gavage and were maintained under the original housing conditions throughout the experiment. The rats in the HS group received daily administrations of equivalent volumes of distilled water via gavage. Subsequently, 1 h later, they were exposed to the HS environment for a duration of 2 h (40 ± 0.2 °C, relative humidity 60–65%) for 8 days. Rats in the SQBE-L, SQBE-M, and SQBE-H groups were administered SQBE via gavage at dosages of 200 mg/kg, 400 mg/kg, and 800 mg/kg, respectively, 1 h prior to each daily HS exposure. Conversely, the control+SBQE group received a high dose of SBQE (800 mg/kg) through gavage but did not undergo HS treatment.

### 2.4. Sample Collection

Twenty-four hours after the final HS exposure, rats were anesthetized with sodium pentobarbital (50 mg/kg, i.p.). Blood samples were collected and centrifuged (3000× *g*, 15 min) to obtain serum; the serum was stored at −80 °C. Liver tissues were harvested; one portion was snap-frozen in liquid nitrogen and stored at −80 °C, while the remainder was fixed overnight in 4% paraformaldehyde, followed by storage in 70% ethanol.

### 2.5. Assessment of Liver Function and Injury

Serum alanine aminotransferase (ALT) and aspartate aminotransferase (AST) activities were quantified using commercial kits (Nanjing Jiancheng Bioengineering Institute, Nanjing, China; Cat# C009-2-1 and Cat# C010-2-1, respectively) according to the manufacturer’s protocols.

### 2.6. Detection of Oxidative Stress Indicators

Liver tissue homogenates were used to quantify oxidative stress markers. Tissue samples (100 mg) were homogenized in 1 mL ice-cold phosphate-buffered saline (PBS, pH 7.4) and centrifuged at 12,000× *g* for 15 min at 4 °C. Supernatants were collected for assays. Protein concentration was determined with a BCA kit (Thermo Fisher, Waltham, MA, USA, Cat# 23227). SOD activity was measured using a WST-8-based kit (Beyotime, Beijing, China; Cat# S0101). CAT activity was detected via the ammonium molybdate method (Nanjing Jiancheng Bioengineering Institute, Nanjing, China; Cat# A007-1-1); GPx activity was analyzed using an NADPH oxidation kit (Sigma-Aldrich, St. Louis, MO, USA, Cat# CGP1). Reduced glutathione (GSH) content and malondialdehyde (MDA) levels were quantified via DTNB and TBA methods, respectively (Beyotime, Beijing, China; Cat# S0053 and Cat# S0131, respectively). All values were normalized to the total protein concentration determined by the BCA assay. Mitochondrial ROS levels were assessed in freshly excised liver tissues using MitoTracker Red CMXRos (CMXRos; Beyotime, Beijing, China; Cat# C1999M). Cryosections prepared within 1 h post-resection were incubated with staining solution (1.2× concentration) at 37 °C for 30 min in the dark. After three 5 min PBS (pH 7.4) washes, the nuclei were counterstained with DAPI, and fluorescent images were obtained using a fluorescence microscope. Mean fluorescence intensity was quantified using ImageJ.

### 2.7. Quantification of Inflammatory Cytokines

Serum and liver tissue homogenates were analyzed for TNF-α, IL-6, IL-1β, and IL-18 levels using commercial ELISA kits (CUSABIO, Wuhan, China; Cat# E11987, Cat# E04640, Cat# E08055, and Cat# E04610, respectively) according to manufacturers’ protocols. Serum cytokine concentrations are expressed as pg/mL. Liver tissue values were normalized to total protein (pg/mg protein) determined by BCA assay.

### 2.8. Histopathological Examination

Following standard hematoxylin and eosin (HE) staining procedures, 4 μm sections from paraffin-embedded liver samples were prepared for general morphology assessment. Three investigators, blinded to treatment assignments, independently scored the degree of inflammation and necrosis using the Ishak system [22].

### 2.9. Detection of Apoptosis

Hepatocyte apoptosis was detected using a commercial TUNEL assay kit (Roche Diagnostics, Basel, Switzerland; Cat# 11684795910), with DAPI counterstaining of nuclei prior to visualization by fluorescence microscopy (Zeiss AX10, Jena, Germany).

### 2.10. Western Blotting Analysis

Total protein was extracted from rat liver tissues using RIPA lysis buffer (Beyotime, Beijing, China, Cat# P0013B) supplemented with protease and phosphatase inhibitors. Protein concentrations were determined by BCA assay. Equal amounts of protein (20 μg) were separated on 10% SDS-PAGE gels and transferred to polyvinylidene difluoride (PVDF) membranes. Membranes were blocked with 5% BSA in TBST for 1 h at room temperature and then incubated overnight at 4 °C with the following rabbit anti-rat monoclonal primary antibodies: Nrf2 (Abcam, Cambridge, UK, Cat# ab137550; 1:1000), HO-1 (CST, Cat# 70081T; 1:1000), NF-κB p65 (Abcam, Cambridge, UK, Cat# ab32536; 1:1000), Phospho-NF-κB p65 (Abcam, Cambridge, UK, Cat# ab239882; Ser536; 1:1000), NLRP3 (Invitrogen, Carlsbad, CA, USA, Cat# PA5-79740; 1:500), and β-actin (Abcam, Cambridge, UK, Cat# ab179467; 1:5000). After TBST washes, membranes were incubated with HRP-conjugated goat anti-rabbit IgG (Abcam, Cambridge, UK, Cat# ab205718; 1:5000) for 1 h at room temperature. Band densities were quantified using ImageJ with normalization to β-actin.

### 2.11. Immunohistochemistry (IHC)

Following standard dewaxing and rehydration, paraffin sections underwent antigen retrieval via heat-induced epitope retrieval in EDTA buffer (pH 8.0; Solarbio, Beijing, China; Cat# C1034) at 95 °C for 20 min. After natural cooling to room temperature, sections were washed thrice in PBS (pH 7.4) for 5 min each under gentle agitation. Endogenous peroxidase activity was subsequently blocked with 3% H_2_O_2_ in PBS for 10 min. Non-specific binding was minimized by incubation in 5% normal goat serum (in PBS) for 1 h at room temperature. Sections were then incubated overnight at 4 °C within a humidified chamber with the following rabbit primary antibodies diluted in PBS: anti-Nrf2 (1:200; Abcam, Cambridge, UK, Cat# ab313825), anti-cleaved caspase-3 (Asp175; 1:50; GeneTex, Irvine, CA, USA, GTX86952), anti-NF-κB p65 (1:200; Abcam, Cambridge, UK, Cat# ab32536), and anti-NLRP3 (1:200; GeneTex, Irvine, CA, USA, GTX100064). After three PBS washes, sections were incubated for 50 min at room temperature with HRP-conjugated goat anti-rabbit IgG secondary antibody (1:1000; Sungene Biotech, Tianjin, China, Cat# LK2001). Antigen–antibody complexes were visualized using DAB substrate (Solarbio, Beijing, China; Cat# DA1010) for 3–5 min, followed by hematoxylin nuclear counterstaining for 5 min. Finally, sections were dehydrated, cleared, and permanently mounted with neutral Canada balsam.

Immunostaining was semi-quantitatively evaluated by integrating scores for staining intensity and the proportion of immunopositive cells. Staining intensity was graded as 0 (negative), 1 (weak), 2 (moderate), or 3 (strong). The percentage of positive cells, quantified using ImageJ software (version 1.8.0; National Institutes of Health, Bethesda, MD, USA), was scored as 0 (<5%), 1 (5–25%), 2 (26–50%), 3 (51–75%), or 4 (>75%). A final IHC score (range 0–12) was calculated for each section by multiplying the intensity score by the proportion score. This assessment was performed on a minimum of six randomly selected high-power fields (400×) per stained section.

### 2.12. Statistical Analyses

Statistical analysis was conducted using SPSS statistics software (version 25.0; IBM Corp., Armonk, NY, USA). Data are expressed as mean ± standard deviation. Normality of all data was assessed using the Shapiro–Wilk test, and homogeneity of variance was confirmed by the Brown–Forsythe test. All datasets were normally distributed and met the assumption of homogeneity of variance; thus, one-way ANOVA with Tukey’s post hoc test was employed for between-group comparisons.

## 3. Results

### 3.1. Extraction Yield of SBQE

The extraction yield of SBQE was determined as 187.26 ± 12.94 mg/g, indicating that 1 g of sprouted black quinoa powder yielded 187.26 ± 12.94 mg of dried extract. This yield was calculated by dividing the dry weight of the final SBQE powder by the initial weight of the sprouted black quinoa powder used for extraction.

### 3.2. SBQE Attenuated HS-Induced Liver Dysfunction and Histopathological Alterations in Rats

**Figure 1:**
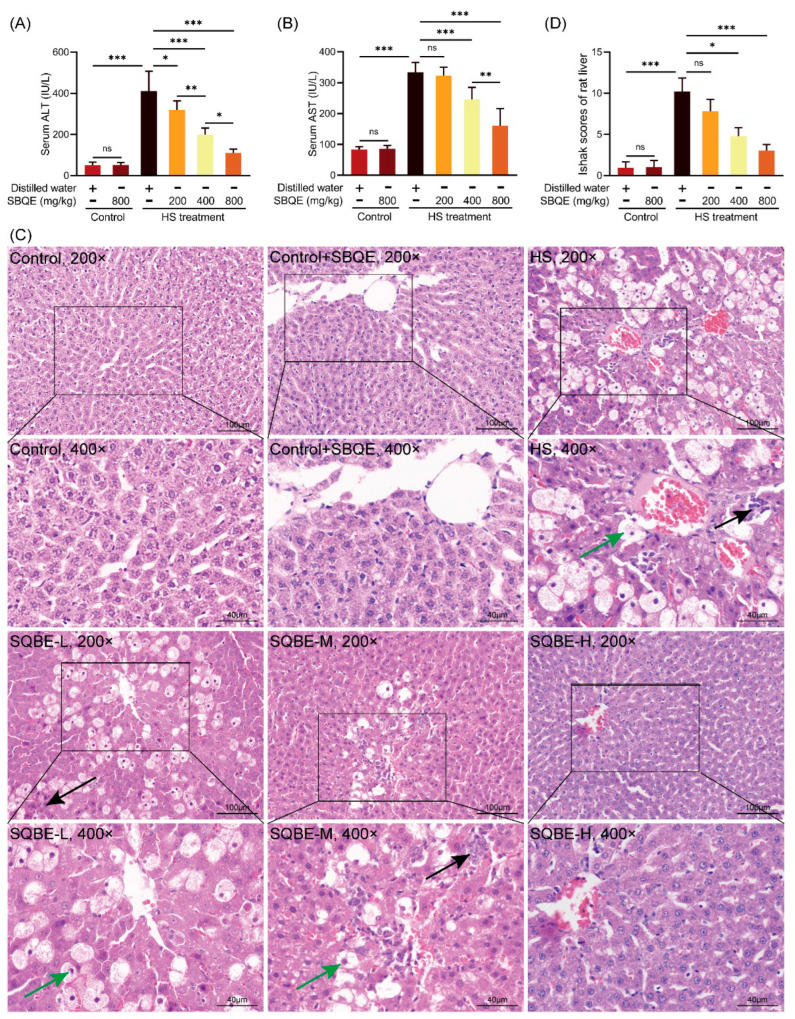
Sprouted black quinoa extract (SBQE) attenuated heat stress (HS)-induced hepatic injury: (**A**) Serum ALT levels in rats of the control, control+SBQE (800 mg/kg), heat stress (HS), and HS+SBQE-treated (200, 400, or 800 mg/kg) groups (*n* = 6). (**B**) Serum AST levels in each group of rats. (**C**) Representative HE-stained liver sections (100× magnification; magnified images were taken at 400× magnification). Green arrows: vacuolization; black arrows: inflammatory infiltration. (**D**) Liver histopathological scores were determined using the Ishak scoring system. Data are expressed as mean ± standard deviation. * *p* < 0.05, ** *p* < 0.01 and *** *p* < 0.001. ns: not significant.

Exposure to HS resulted in notable liver injury, as demonstrated by significantly elevated serum ALT (Figure 1A) and AST (Figure 1B) levels in comparison to the control group. Pretreatment with SBQE mitigated this elevation in a dose-dependent manner, with the high dose (800 mg/kg) exhibiting significant differences relative to the HS group (*p* < 0.001). Histopathological evaluation (Figure 1C,D) supported the biochemical findings. Control rats displayed normal hepatic histology characterized by intact lobular architecture, distinct hepatocyte cell borders, and an absence of pathological alterations. In contrast, livers from the HS group showed severe pathological changes, including pronounced hepatocyte vacuolization (indicated by green arrows), disorganized hepatocyte arrangement, loss of hepatic cords and sinusoids, and infiltration of inflammatory cells (indicated by black arrows). These alterations resulted in significantly higher histopathological scores compared to the control group. Correspondingly, SBQE intervention significantly ameliorated these histopathological lesions in a dose-dependent manner. At a dose of 800 mg/kg, SBQE restored hepatic architecture compared to the HS group, with minimal vacuolization and infrequent inflammatory foci. Importantly, the administration of SBQE at a dosage of 800 mg/kg, in the absence of HS, did not result in any abnormalities in serum markers, nor in histological assessments, thereby affirming the safety profile of SBQE.

### 3.3. SBQE Mitigated Hepatic Oxidative Stress Caused by HS in Rats

**Figure 2:**
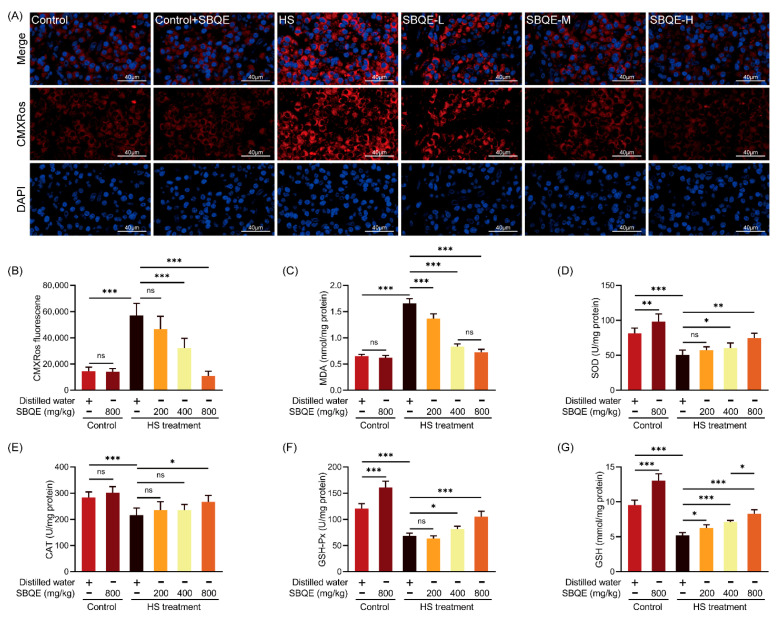
Sprouted black quinoa extract (SBQE) mitigated heat stress (HS)-induced hepatic oxidative stress: (**A**) Representative fluorescence images of mitochondrial ROS (mROS; red: MitoTracker Red CMXRos; blue: DAPI nuclei). (**B**) Quantification of mitochondrial ROS fluorescence intensity. (**C**) Malondialdehyde (MDA) content in liver tissues. (**D**–**F**) Activities of antioxidant enzymes: superoxide dismutase (SOD), catalase (CAT), and glutathione peroxidase (GSH-Px). (**G**) Reduced glutathione (GSH) content. Data are expressed as mean ± standard deviation. * *p* < 0.05, ** *p* < 0.01 and *** *p* < 0.001. ns: not significant.

HS induced significant oxidative damage in hepatic tissues, as evidenced by a pronounced increase in mitochondrial ROS (mROS) fluorescence intensity (Figure 2A,B) and a notable accumulation of MDA (Figure 2C) when compared to control rats. Concurrently, the liver tissues of rats in the HS group exhibited a marked reduction in the activity of crucial antioxidant enzymes, including SOD (Figure 2D), CAT (Figure 2E), and GSH-Px (Figure 2F), along with a depletion of GSH reserves (Figure 2G). Pretreatment with SBQE mitigated the alterations induced by HS in a dose-dependent manner. Administration of a high dose of SBQE (800 mg/kg) resulted in the reversal of all assessed parameters, with mROS fluorescence intensity and MDA levels comparable to those of the control group (*p* > 0.05), significantly increasing the activities of SOD, CAT, and GSH-Px, as well as the GSH reserves. Importantly, rats treated with SBQE alone (control+SBQE) demonstrated significantly enhanced activities of key antioxidant enzymes (SOD, CAT, and GSH-Px) compared to controls, while maintaining baseline mROS levels and physiological GSH content. This illustrates SBQE’s dual ability to enhance intrinsic antioxidant defenses while avoiding metabolic disturbances, thereby affirming its potential prophylactic efficacy.

### 3.4. SBQE Inhibited the Hepatic Inflammatory Response Elicited by HS in Rats

**Figure 3:**
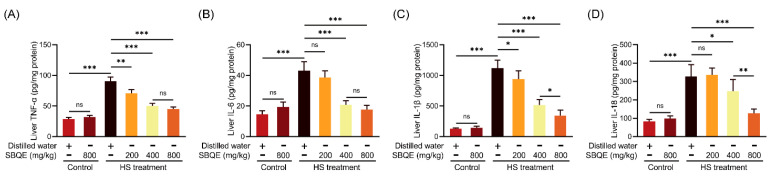
Sprouted black quinoa extract (SBQE) suppressed heat stress (HS)-induced hepatic inflammation. Cytokines quantified in liver homogenates by using the ELISA assay, normalized to total protein: (**A**) The content of tumor necrosis factor-alpha (TNF-α) in rat liver. (**B**) Liver interleukin-6 (IL-6) content. (**C**) Liver interleukin-1β (IL-1β) content. (**D**) Liver interleukin-18 (IL-18) content. Data are expressed as mean ± standard deviation. * *p* < 0.05, ** *p* < 0.01 and *** *p* < 0.001. ns: not significant.

HS induced a pronounced hepatic inflammatory response, resulting in a significant elevation of proinflammatory cytokine levels within liver tissues, specifically TNF-α (Figure 3A), IL-6 (Figure 3B), IL-1β (Figure 3C), and IL-18 (Figure 3D), when compared to unstressed control groups. Pretreatment with SBQE attenuated these increases in a dose-dependent manner, with high-dose SBQE reducing IL-6 and IL-18 levels to values statistically comparable to baseline controls. Importantly, rats administered SBQE alone exhibited cytokine profiles that were indistinguishable from those of the control group, indicating the absence of intrinsic immunotoxicity.

### 3.5. SBQE Activated the Nrf2 Antioxidant Signaling Pathway in the Liver of HS Rats

**Figure 4:**
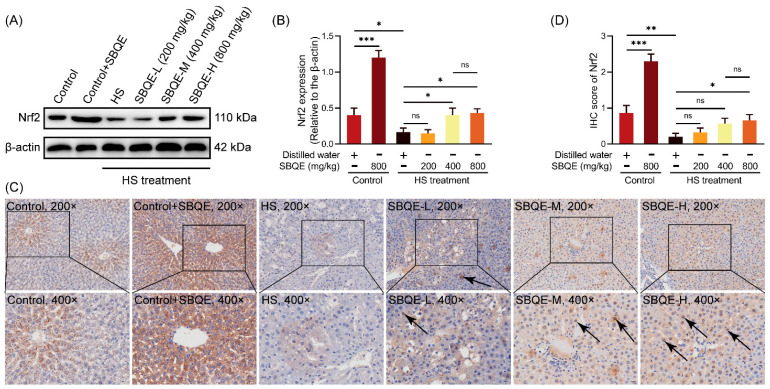
Sprouted black quinoa extract (SBQE) activated the Nrf2 signaling pathway in the liver of rats subjected to heat stress (HS): (**A**) Representative Western blotting analysis of hepatic Nrf2 protein expression in rats. (**B**) Quantification of total Nrf2 protein levels normalized to β-actin. (**C**) Immunohistochemical (IHC) staining of liver sections. (**D**) IHC scores of Nrf2 expression. Data are expressed as mean ± standard deviation. * *p* < 0.05, ** *p* < 0.01 and *** *p* < 0.001. ns: not significant.

SBQE exhibited a notable influence on the hepatic Nrf2 signaling pathway in rats under both basal and HS conditions. In non-stressed rats, administration of SBQE led to a substantial increase in total Nrf2 protein expression compared to the control group, as verified by Western blotting analysis (Figure 4A,B) and elevated IHC scores (Figure 4C,D). However, this upregulation did not coincide with observable nuclear translocation in the liver under resting physiological conditions. Under HS conditions, SBQE pretreatment resulted in a dose-dependent nuclear accumulation of Nrf2 in hepatocytes. This was demonstrated by progressively intensified nuclear immunostaining patterns across treatment groups: the medium-dose SBQE (400 mg/kg) exhibited moderate but discernible nuclear enrichment, whereas the high dose (800 mg/kg) showed pronounced nuclear translocation, significantly exceeding the levels observed in the HS group. IHC quantification corroborated this dose-responsive increase, revealing incrementally higher nuclear Nrf2 immunoreactivity scores with increasing SBQE concentrations. Statistically significant nuclear translocation was observed exclusively at the highest dose compared to HS rats. This biphasic mechanism, which enhances Nrf2 protein synthesis at the basal level without inducing premature nuclear translocation and yet facilitates stress-responsive nuclear accumulation in a dose-dependent manner, demonstrates SBQE’s ability to prime the rat’s antioxidant defense system while preserving the physiological regulation of Nrf2 activation kinetics.

### 3.6. SBQE Effectively Suppressed the Activation of NF-κB and the NLRP3 Inflammasome Pathway Induced by HS

**Figure 5:**
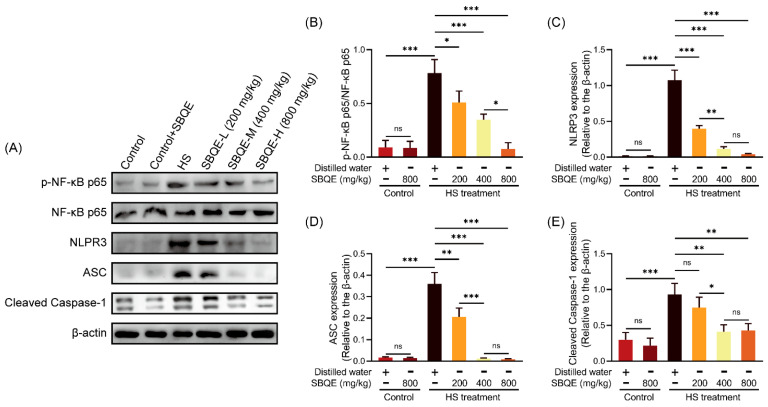
Sprouted black quinoa extract (SBQE) inhibited heat stress (HS)-induced NF-κB activation and NLRP3 inflammasome assembly: (**A**) Representative Western blotting of NF-κB pathway and NLRP3 inflammasome components: phospho-NF-κB p65 (p-NF-κB p65), total NF-κB p65, NLRP3, apoptosis-associated speck-like protein containing a CARD (ASC), cleaved caspase-1, and β-actin. (**B**) Quantification of p-NF-κB p65/ NF-κB p65 ratio. (**C**–**E**) Quantitative analysis of NLRP3, ASC, and cleaved caspase-1 protein levels normalized to β-actin. Data are expressed as mean ± standard deviation. * *p* < 0.05, ** *p* < 0.01 and *** *p* < 0.001. ns: not significant.

This study demonstrated that HS robustly activated the NF-κB signaling pathway and the NLRP3 inflammasome in rat liver tissue, as evidenced by a significant increase in phospho-NF-κB p65 levels (Figure 5A,B) and the upregulation of NLRP3 (Figure 5A,C), ASC (Figure 5A,D), and cleaved caspase-1 (Figure 5A,E) expression in comparison to unstressed control groups. Pretreatment with SBQE resulted in a dose-dependent suppression of this inflammatory cascade. Specifically, the accumulation of phospho-NF-κB p65 was progressively attenuated with increasing doses of SBQE, with the highest dose (800 mg/kg) reducing phosphorylation to levels statistically indistinguishable from the control group (*p* > 0.05). Furthermore, SBQE administration led to a dose-responsive reduction in the expression of NLRP3 and ASC proteins and significantly decreased the generation of activated cleaved caspase-1, thereby effectively impeding inflammasome assembly and its effector functions. Importantly, rats in the control+SBQE group maintained baseline expression levels of all inflammatory markers, indicating that SBQE selectively inhibited HS-induced activation without affecting the constitutive immunocompetence of the subjects.

### 3.7. SBQE Suppressed HS-Induced Hepatocellular Apoptosis in Rats

**Figure 6:**
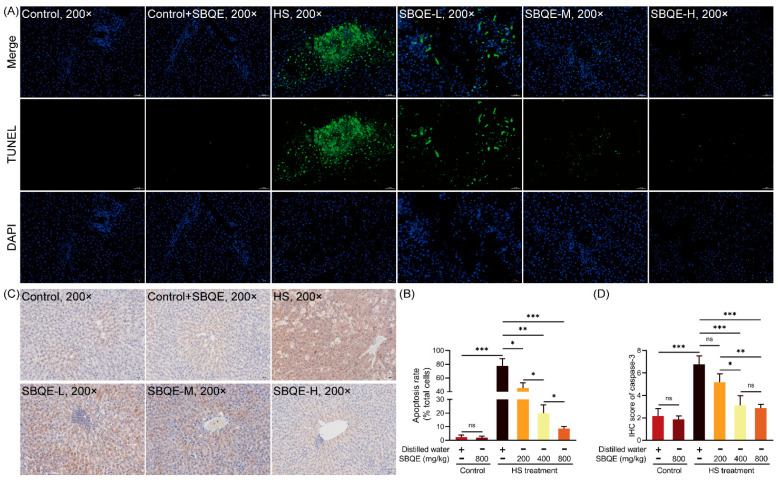
Sprouted black quinoa extract (SBQE) suppressed heat stress (HS)-induced hepatocellular apoptosis: (**A**) Representative TUNEL staining (green: apoptotic nuclei; blue: DAPI counterstain). (**B**) Apoptosis index (TUNEL⁺ nuclei/total nuclei %). (**C**) Cleaved caspase-3 immunohistochemistry (brown: positive signal; hematoxylin counterstain). (**D**) Quantification of cleaved caspase-3 immunoreactivity by mean optical density (MOD). Data are expressed as mean ± standard deviation. * *p* < 0.05, ** *p* < 0.01 and *** *p* < 0.001. ns: not significant.

HS induced significant hepatocellular apoptosis in rats, as demonstrated by a high prevalence of TUNEL-positive nuclei, indicative of DNA fragmentation (Figure 6A), and increased immunoreactivity for cleaved caspase-3 (Figure 6C) compared to minimal baseline levels observed in control groups. Pretreatment with SBQE resulted in a dose-dependent reduction in these apoptotic markers. Quantitative analysis of TUNEL staining (Figure 6B) showed a progressive dose-dependent reduction in apoptosis indices. The 800 mg/kg SBQE group showed no statistically significant difference in apoptosis index compared to the control group (*p* > 0.05). Similarly, cleaved caspase-3 immunohistochemistry (Figure 6D) revealed a dose-dependent decrease in cytoplasmic staining intensity, with densitometric analysis indicating maximal suppression at doses of 400 or 800 mg/kg. Importantly, rats in the control+SBQE group exhibited TUNEL positivity and caspase-3 activation comparable to control levels, thereby confirming the pharmacological specificity of SBQE in mitigating HS-induced apoptosis without exerting intrinsic pro-apoptotic effects.

## 4. Discussion

The present study offers robust evidence that SBQE exerts significant hepatoprotective effects against liver injury induced by HS. This conclusion is supported by the dose-dependent reduction in serum ALT and AST levels—enzymes typically sequestered within hepatocytes that are released into the bloodstream upon plasma membrane disruption, serving as sensitive indicators of hepatocellular damage [23,24]. Additionally, there was a marked reduction in histopathological lesions, including hepatocyte vacuolization and inflammatory infiltration. These enhancements in hepatic structure and function are mechanistically attributed to SBQE’s ability to interrupt the pathological progression from oxidative stress to inflammation and apoptosis, a cascade that characterizes HS-induced organ failure [1]. Notably, the safety of SBQE at the tested doses (200, 400, and 800 mg/kg) was validated in the control+SBQE group, where no abnormalities in serum ALT/AST levels or hepatic histology were observed, confirming the absence of intrinsic hepatotoxicity. This aligns with previous studies: high-dose red quinoa bran extracts did not induce hepatotoxicity in mice but rather alleviated liver injury [17]. Collectively, these findings support the safety of the SBQE doses used in this study.

The protective effect observed was primarily attributed to the reduction in mitochondrial oxidative stress, a key factor in the pathogenesis of HS [6,25]. Pretreatment with SBQE significantly inhibited the accumulation of mROS and lipid peroxidation, as indicated by MDA levels. Additionally, it restored the activities of essential antioxidant enzymes, including SOD, CAT, and GSH-Px, as well as replenished GSH reserves. Research indicates that the antioxidant properties of quinoa are primarily ascribed to its substantial concentration of bioactive compounds, including polyphenols, saponins, and flavonoids [26]. Numerous studies have demonstrated that dark quinoa varieties, such as black quinoa, possess higher levels of total polyphenols and flavonoids, resulting in enhanced antioxidant activity compared to other colored quinoa varieties [14,27]. Extracts derived from black quinoa varieties have been shown to effectively mitigate oxidative stress in HMEC-1 cells [28]. Recent studies have further indicated that the antioxidant properties and polyphenol levels in quinoa are strongly linked to the variety, with the darkest types showing the best bioactive characteristics, irrespective of their region of origin [29]. Moreover, the germination of quinoa has been shown to enhance the concentration of polyphenols and flavonoids, thereby increasing its antioxidant activity [30,31,32]. Previous research has demonstrated that sprouted quinoa offers significant benefits, increasing polyphenols from 1.5 to 2.5 mg GaE/g dry weight (DW) and flavonoids from 3 to 4.5–5.0 mg QE/g DW. It contains more macro/microelements and is non-genotoxic while protecting against ROS-induced damage [15]. These phytochemicals have been extensively documented to exhibit antioxidant and anti-inflammatory properties, which are critical mechanisms in alleviating liver injury induced by HS. Similarly, compared with the seed extract, the germinated quinoa extract shows increased total phenolic content and flavonoids, as well as enhanced antioxidant capacity [33]. Al-Qabba et al. [34] reported similar results: quinoa sprout extracts significantly lowered MDA levels and effectively boosted the reduced GSH and SOD activities in rats subjected to oxidative stress. Overall, the superior antioxidant activity of SBQE is the primary factor contributing to its efficacy in mitigating oxidative damage to the liver induced by HS.

The Nrf2-Keap1 signaling pathway plays a crucial role in defending against HS-induced oxidative stress [4,10,21]. Under normal conditions, Nrf2 is associated with Keap1, which holds Nrf2 in the cytoplasm. However, under oxidative stress, Keap1 is inactivated, which results in Nrf2 translocation to the nucleus and activates the ARE-mediated transcriptional response [8]. Earlier research indicates that quinoa water extract boosts antioxidant capacity by triggering the Nrf2 signaling pathway, which in turn reduces bleeding points and areas in rats with ethanol-induced gastric mucosal damage [35]. SBQE enhances the expression and nuclear translocation of Nrf2 during HS, thereby promoting the transcriptional upregulation of cytoprotective genes responsible for encoding antioxidant enzymes. Importantly, the absence of Nrf2 translocation in SBQE-treated rats under non-stress conditions suggests a context-dependent activation mechanism, thereby preventing unnecessary metabolic expenditure in basal states.

Inflammation is a crucial response to oxidative stress, and it can also, in turn, enhance the progression of oxidative stress [8]. Quinoa, a gluten-free grain, not only avoids eliciting inflammatory responses but also demonstrates significant anti-inflammatory properties in both in vitro and in vivo studies [36]. It has been reported that saponins are the primary antinutritional elements in quinoa, and they have notable anti-inflammatory properties, reducing the release of inflammatory cytokines like TNF-α and IL-6 in lipopolysaccharide-stimulated RAW264 cells [37]. Lin et al. [38] showed that saponins in quinoa bran reduced kidney inflammation and damage in mice with high uric acid levels by blocking PI3K/AKT/NF-κB inflammatory signaling. The administration of extracts derived from sprouted red and yellow quinoa seeds to CCL4-induced rat models resulted in a significant reduction in hepatic inflammation [34]. This study offers additional evidence indicating that SBQE significantly attenuates liver inflammatory cascades induced by HS. Hepatic levels of proinflammatory cytokines (TNF-α, IL-6, IL-1β, and IL-18) were markedly reduced, coinciding with inhibition of the NF-κB/NLRP3 inflammasome axis. Specifically, SBQE attenuated the phosphorylation of NF-κB p65 and suppressed the expression of NLRP3, ASC, and cleaved caspase-1. Similarly, this dual inhibition likely stems from triterpenoid saponins abundant in black quinoa—such as phytolaccagenic acid and hederagenin—which disrupt IκB kinase (IKK) activation and ASC oligomerization [37,38]. Moreover, the consequent reduction in mature IL-1β/IL-18 not only curtailed neutrophil recruitment but also interrupted the ROS-NF-κB positive feedback loop that perpetuates inflammation [8].

The convergence of oxidative and inflammatory insults culminated in hepatocyte apoptosis, as evidenced by increased TUNEL⁺ nuclei and cleaved caspase-3 immunoreactivity. SBQE pretreatment substantially attenuated these apoptotic markers, suggesting interference with intrinsic (mitochondrial) and/or extrinsic (death receptor) pathways. This anti-apoptotic activity may be attributed to quinoa-derived bioactive peptides (BAPs), which have been identified in sprouted grains through peptidomic analyses. These BAPs potentially stabilize mitochondrial membranes, thereby preventing the release of cytochrome C, and directly inhibit the activation of caspase-3 [27,39].

## 5. Conclusions

SBQE attenuated HS-induced liver injury by activating Nrf2-mediated antioxidant defenses and suppressed NF-κB/NLRP3-driven inflammation and apoptosis. The dose-dependent efficacy, safety profile, and germination-enhanced bioactivity position SBQE as a promising natural intervention for HS-related liver injury. Future research should aim to identify and isolate the specific active compounds responsible for the activation of Nrf2 and the inhibition of NF-κB/NLRP3. These findings suggest the health attributes of SBQE in reducing HS-induced liver injury due to the comprehensive effect of functional components, such as flavonoids, polyphenols, saponins, and other nutrients. Future research should focus on clinical translation in heat-stressed livestock/poultry and isolating active compounds for nutraceutical development.

## Data Availability

The original contributions presented in the study are included in the article; further inquiries can be directed to the corresponding author.

## Abbreviations

Nrf2	Nuclear factor erythroid 2-related factor 2
NF-κB	Nuclear factor-κB
NLRP3	NOD-like receptor pyrin domain-containing 3
HS	Heat stress
SBQ	Sprouted black quinoa
SBQE	Sprouted black quinoa extract
ALT	Alanine aminotransferase
AST	Aspartate aminotransferase
TNF-α	Tumor necrosis factor-alpha
IL	Interleukin
ASC	Apoptosis-associated speck-like protein containing a CARD
ROS	Reactive oxygen species
Keap1	Kelch-like ECH-associated protein 1
ARE	Antioxidant response element
SOD	Superoxide dismutase
CAT	Catalase
GSH	Reduced glutathione
GSH-Px	Glutathione peroxidase
MDA	Malondialdehyde
IHC	Immunohistochemistry
